# In silico nephroprotective evaluation of microbial biotransformed metabolites from *Aframomum melegueta*

**DOI:** 10.1186/s13568-025-01962-x

**Published:** 2025-10-22

**Authors:** Rabab Mahrous Abdou, Riham Salah El-Dine, Reham Samir, Nebal Darwish El-Tanbouly, Aly Mohamed El-Sayed

**Affiliations:** 1 Department of Pharmacognosy, Faculty of Pharmacy, Cairo University, Cairo, Egypt; 2 Department of Microbiology and Immunology, Faculty of Pharmacy, Cairo University, Cairo, Egypt

**Keywords:** Biotransformation, *Aframomum melegueta*, Nephroprotective activity, Gingerol, Shogaol, Paradol

## Abstract

Microbial biotransformation of three bioactive phenolic constituents from *Aframomum melegueta* K. schum, namely 6-gingerol, 6-paradol and 6-shogaol, was performed by *Bacillus subtilis* 168, *Pseudomonas aeruginosa* PO1A and *Candida albicans* ATCC10231. Structures of the isolated compounds were determined using LC/MS analyses*.* To assess and compare their potential nephroprotective effects, the parent compounds and their biotransformation metabolites were subjected to molecular docking studies targeting AMP-activated protein kinase (AMPK) for the first time. During microbial biotransformation, a series of reactions, primarily hydroxylation and reduction, were observed, resulting in the identification of five distinct metabolites. LC/MS analysis of the fermentation medium revealed that *Bacillus subtilis* 168 converted 6-gingerol into 6-gingerdiol (M1) and hydroxylated 6-gingerol (M2), while 6-shogaol was transformed into 6-paradol (M3) and hydroxylated 6-shogaol (M4). Additionally, 6-paradol underwent further reduction to form (M5). Docking results showed that all compounds demonstrated binding affinity to AMPK, indicating potential nephroprotective activity. Notably, M1 exhibited the highest binding affinity, suggesting its strong therapeutic promise as a nephroprotective agent of natural origin. M5 ranked second in binding affinity, followed by M4. These results highlight the effectiveness of microbial transformation in generating bioactive derivatives with potentially enhanced biological activity compared to their natural precursors.

**Supplementary Information:**

The online version contains supplementary material available at 10.1186/s13568-025-01962-x.

## Introduction

Microbial transformation is recognized as a promising green technique for preparing analogues of bioactive natural compounds (Alfarra and Omar 2012; Venisetty and Ciddi 2005). It has been extensively used to produce new and therapeutically valuable metabolites of almost all classes, *i.e.* steroids, terpenes, phenolics (Shah et al. 2014). In both laboratory and industrial scale, microbial transformation provides more suitable alternative for chemical synthesis because of mild operating conditions and lower cost. Moreover, microorganisms can produce a great variety of enzymes in a short period of time and provide stereoselective modification of complex structures.

Phenolic compounds are phytochemical class, which are widely distributed in most plant species and possess numerous bioactive properties. *A. melegueta* K. schum phenolics (6-gingerol, 6-shogaol and 6-paradol) have been reported to exhibit several pharmacological activities such as anti-inflammatory, antioxidant, anticancer, analgesic, antiemetic and neuroprotective effects (Tian et al. 2020; Sapkota et al. 2019; Kou et al. 2018; Seow et al. 2017; Gaire et al. 2015; Wang et al. 2014; Young et al. 2005). Modifying the structure of simple phenolics with the aim of finding new metabolites with higher therapeutic potential was the subject of several biotransformation studies. Microbial catalytic reactions could affect either the aromatic ring or the side chain. Previous reports have documented the biotransformation of 6-gingerol, 6-shogaol (Koh and Lee 1983; Takahashi et al. 1993).

The studies revealed that 6-shogaol is more effective than 6-gingerol as antioxidant, anti-inflammatory and anticancer compound (Dugasani et al. 2010; Guo et al. 2014; Pan et al. 2008). This was illustrated by the *α,β*-unsaturated ketone moiety which confers an excellent potency and efficacy to 6-shogaol (Guo et al. 2014). Also, 6-shogaol exhibits greater DPPH radical scavenging capacity and ferric reducing antioxidant ability than its homologs 8- and 10-shogaol as the length of the alkyl chain plays a role in the antioxidant effect of shogaols (Guo et al. 2014). Therefore, structural modification of these bioactive compounds may generate new chemical derivatives with greater biological activities and microbial transformation could be an effective tool for this purpose.

Recently, informatics and computational biology have been developed to facilitate the process of drug discovery. Molecular docking is one of the most popular approaches that has experienced significant progress in predicting biological activities of natural products (Kumara et al. 2017).

In an effort to discover new biologically active metabolites, the inherent ability of three microorganisms; namely *Bacillus subtilis* 168, *Candida albicans* ATCC10231 and *Pseudomonas aeruginosa* PO1A to change the structure of three *A. melegueta* phenolics were evaluated in this study. Additionally, an in-silico aimed at finding out whether the structural modification of 6-paradol, 6-gingerol and 6-shogaol by microbial biotransformation led to improving their activities or not.

## Material and methods

### Chemicals and microorganisms

Yeast extracts microbiological grade, peptone microbiological grade and Sabauraud dextrose agar were obtained from Lab M Ltd, UK. Nutrient agar was purchased from Oxoid Ltd, England. Sucrose and glucose were purchased from El Gomhouria Company for Trading Pharmaceutical Chemicals Medical Appliances, Egypt. Methanol and acetonitrile for LC-ESI/Triple TOF/MS analysis were obtained from Fisher Scientific, UK. Ultrapure water was obtained using the Millipore Milli-Q system (Millipore Corp., USA). All other chemicals were of the highest available analytical grade.

Two bacterial strains; Bacillus *subtilis* 168 and *Pseudomonas aeruginosa* PAO1 and one fungal strain; *Candida albicans* ATCC 10231 were supplied by the department of Immunology and Microbiology, Faculty of Pharmacy, Cairo University. *B. subtilis* and *P. aeruginosa* were cultivated on nutrient agar at 37 °C for 24 h and *C. albicans* were cultivated on Sabauraud dextrose agar at 30 °C for 48 h.

### Plant material and isolation of the parent compounds

*Aframomum melegata* K. schum seeds were purchased from an herbal store (Haraz, Cairo, Egypt) and were identified by Prof. Abdelhaleem A. Mohamed, from the Flora and Phytotaxonomy Research Department, of the Agricultural Museum (Dokki, Egypt). A voucher specimen (No. 3.7.2019) was deposited in the Herbarium of the Pharmacognosy Department, Faculty of Pharmacy, Cairo University. 1.5 kg of *A. melegata* powdered seeds were extracted with methanol (3 × 3L) using an Ultraturrax blender, and the combined methanol extracts were evaporated using a rotatory evaporator under reduced pressure and at a temperature not exceeding 50 °C to provide 78 g of *A. melegata* seeds extract. 6-gingerol, 6-shogaol, and 6-paradol were isolated and identified according to the procedures described by El-Halawany et al. 2014.

### Biotransformation procedures

A culture medium for microorganisms was prepared by dissolving glucose (5 g), yeast extract (5 g), peptone (1 g), sucrose (2 g) in distilled water (4.0 L). The medium was sterilized at 121 °C for 15 min. Fermentations were accomplished according to a standard two-stage protocol (Gentile et al. 1996; Cichewicz and Kouzi 1998). The cultivated microorganism was suspended in 50 mL of liquid medium in 250 mL Erlenmeyer flask and the flask was shaken (reciprocating shaker, 100 rpm) at 30 °C for 24 h. After 24 h incubation, 10 mL of the precultured medium was transferred into the 250-mL Erlenmeyer flask containing 40 mL of fresh medium and was shaken (100 rpm) for an additional 24 h. After the growth of microorganisms, 10 mg of the parent compound (50 mg dissolved in 0.5 mL DMSO) was added to the medium. Positive/substrate controls consisted of autoclaved medium incubated with the substrate without microorganisms. Negative/culture controls consisted of medium in which microorganisms were grown under identical conditions without addition of the substrate. Cultures were sampled at days 1, 3, 5, 7 and 14 by extracting the broth with ethyl acetate. Ethyl acetate (5 mL) was added to the reaction sample (5 mL), mixed for 2 min using vortex and centrifuged for 5 min. The upper layer was collected, evaporated to dryness under reduced pressure at 40 °C and were examined by thin layer chromatography on precoated silica gel sheets then dissolved in HPLC-grade methanol (100 μg/mL), filtered using a membrane disc filter (0.2 μm) and analyzed via LC-TQ/MS.

### LC-TQ/MS analysis

The samples were analyzed via LC-TQ/MS adopting the technique described (Supplementary file) in negative ion mode.

### Docking study of the parent compounds and metabolites to the binding site of AMKP

The parent compounds (6-gingerol, 6-shogaol and 6-paradol) and their metabolites M1-M5 were docked into the binding site of AMKP to predict and compare their nephroprotective activities.

The molecular modeling studies were carried out using molecular operating environment (MOE, 2019.0102) software. All minimizations were performed with MOE until an RMSD gradient of 0.1 kcal mol^−1^ Å^−1^ with MMFF94x force field and the partial charges were automatically calculated. The X-ray crystallographic structure of AMP-Activated Protein Kinase (AMPK) co-crystalized with 2-({5-bromo-2-[(3,4,5-trimethoxyphenyl)amino]pyrimidin-4-yl}oxy)-N-methylbenzene-1-carboximidic acid (EDJ) (PDB ID: 6BX6) was downloaded from the protein data bank (https://www.rcsb.org/structure/6BX6). For each co-crystallized enzyme, water molecules and ligands which were not involved in the binding were removed, the protein was prepared for the docking study using *Protonate 3D* protocol in MOE with default options. The co-crystalized ligand (EDJ) was used to define the binding site for docking. Triangle matcher placement method and London dG scoring function were used for docking. The co-crystallized ligand EDJ was used to define the binding site for docking.

## Result

### LC-TQ/MS analysis of the biotransformed products

**Fig. 1:**
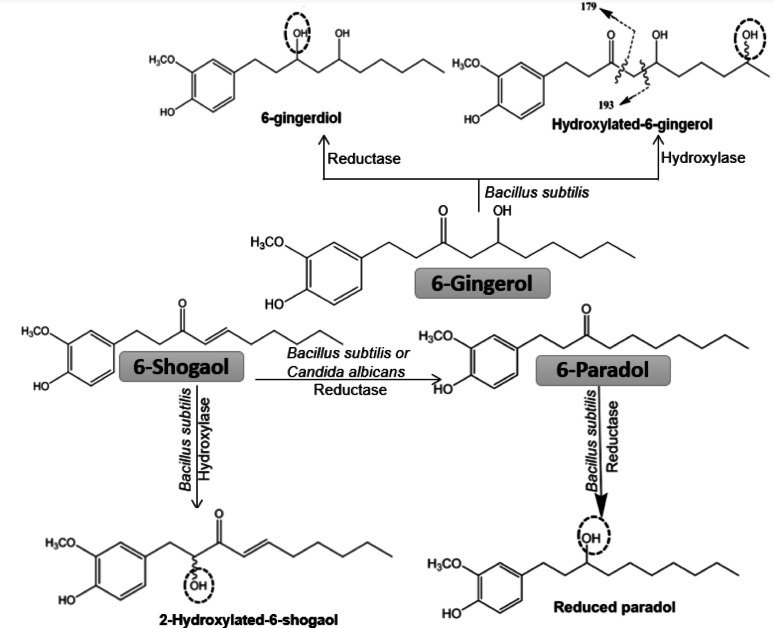
The biotransformed metabolites of 6-gingerol, 6-shogaol and 6-paradol with *B. subtilis* 168 and *C. albicans* ATCC 10231 and the proposed microbial enzymatic reactions

TLC monitoring during the fragmentation process enabled the detection of multiple metabolites (Fig. S1–S4). In total, five metabolites were identified in the reaction mixtures of *B. subtilis* treated with *A. meleguata* phenolics (Fig. 1). Their chemical structures were elucidated based on high-resolution mass spectrometry data and interpretation of MS/MS fragmentation patterns (Fig. S5–S7).

*B. subtilis* was able to biotransform 6-gingerol as two major peaks at 13.91 min (M1) and 13.15 (M2) were observed (Fig. S5). M1 was found as deprotonated ion [M + H]^−^ at *m/z* 295.234 with two mass units higher than that of 6-gingerol (*m/z* 293), which can be associated with the reduction of the keto group into hydroxyl group. The MS–MS spectrum of M1 (Fig. S5B) showed base peak at *m/z* 195 further suggests presence of a hydroxyl group on C3. Therefore, M1 was tentatively identified as 6-gingerdiol (Abdou et al. 2021).The MS–MS spectrum of M2 (Fig. S5C) revealed molecular ion at *m/z* 309.4179, indicating a mass increase of 16 Da compared to the parent compound, 6-gingerol. This mass difference suggests hydroxylation of the starting substrate. Similar to 6-gingerol, M2 produced fragment at *m/z* 193 likely due to cleavage between C4–C5 (Abdou et al. 2021). Such fragmentation pattern implies that the additional hydroxyl group is located on the alkyl chain beyond C5. However, no other obtained fragment could confirm the precise position of the additional hydroxyl group. The earlier elution of M2 compared to M1 suggests a more polar structure, which supports the presence of the hydroxyl group and aids in its identification. Notably, a metabolite with the same molecular ion was previously isolated from the biotransformation of 6-gingerol with *Aspergillus niger* and was identified as 9-hydroxylated 6-gingerol (Takahashi et al. 1993).

Fermentation of 6-shogaol with *B. subtilis* produced two major metabolites at 24.16 min (M3) and 18.63 min (M4), Fig. S6*.* The MS–MS spectrum of M3 (Fig. S6B) revealed molecular ion and base peak at *m/z* 277.3585 and 141.1502, respectively, which support the identification of M3 as 6-paradol. The peak at 8.63 min (M4) showed molecular ion at *m/z* 291.2326, suggesting addition of hydroxyl group (16 Da) to the parent compound. Furthermore, base peak at *m/z* 155.0441 (Fig. S6C) indicated that the modification at the side chain. The product ions at *m/z* 57 (C_4_H_9_) and 71 (C_5_H_11_) suggest the hydroxylation probably occurred at C2. Consequently, M4 was tentatively identified as 2-hydroxy-1-(4-hydroxy-3-methoxyphenyl) dec-4-en-3-one. However, further evidence is required to confirm the hydroxylation site.

6-Paradol was found as a deprotonated ion at m/z *m/z* 277.1289 (Fig S7A). Further fragmentation revealed an abundant product ion at 141 m*/z* indicating that this peak is the starting material 6-paradol. One metabolite, more polar than the 6-paradol (M5), appeared after 24 h of fermentation with *B. subtilis* and disappeared at day 3 MS/MS spectrum of M5 (Fig. S7B) showed molecular ion at *m/z* 279.0458 and major fragment at *m/z* 143. A mass difference of 2 Da between the molecular and product ion of M5 and those of the parent 6-paradol suggests the reduction of the carbonyl group into a hydroxyl group. Furthermore, the shorter retention time of M5 compared to 6- paradol supports the biotransformation toward a more polar form. Consequently, M5 was tentatively identified as reduced 6-paradol (Lee and Lee 1995).

Fermentation of 6-shogaol *with C. albicans* led to production of a metabolite after 24 h of cultivation (Fig. S4). By time the parent compound disappeared and only the metabolite was detected in the medium at the fifth day.

The metabolite was suggested to be 6-paradol by comparing its R_f_ with that of 6-paradol previously isolated in this study. Thus, biotransformation of 6-shogaol with *C. albicans* led to reduction of the *α, β*-unsaturated ketone to a saturated ketone and accumulation of 6-paradol.

### Docking study to the binding site of AMKP

Docking setup was first validated by self-docking of the co-crystallized ligand (EDJ) in the vicinity of the binding site of the enzyme, the docking score (S) was − 9.7184 kcal/mol and root mean square deviation (RMSD) was 2.5198 Å. Through examination of the binding interactions of EDJ to the active site of the enzyme, it shows H-bond interactions with Val96, halogen bond with Met93 and electrostatic interactions with Val96 and Gllu94 (Fig. S8). The binding energy value (Table 1) is related to the amount of energy that is required by the ligand to bind to a receptor. The less the binding energy (more negative value), the more stable the bond between ligand and receptor. Type of interactions and bond length of the investigated compounds with amino acids of AMP-activated protein kinase were presented in table S1.

**Table 1:** Binding scores (Kcal/mol) of *A. melegueta* K. schum phenolics and their metabolites with amino acids of AMP-activated protein kinase

Compound	Docking score (kcal/mol)
EDJ	− 9.7184
6-Gingerol	− 9.4901
6-Gingerdiol (M1)	− 10.6344
Hydroxylated-6-Gingerol (M2)	− 9.4555
Shogaol	− 8.8991
Hydroxylated-6-Shogaol (M4)	− 8.7352
6-Paradol (M3)	− 8.9965
Reduced Paradol (M5)	− 9.5722

**Fig. 2:**
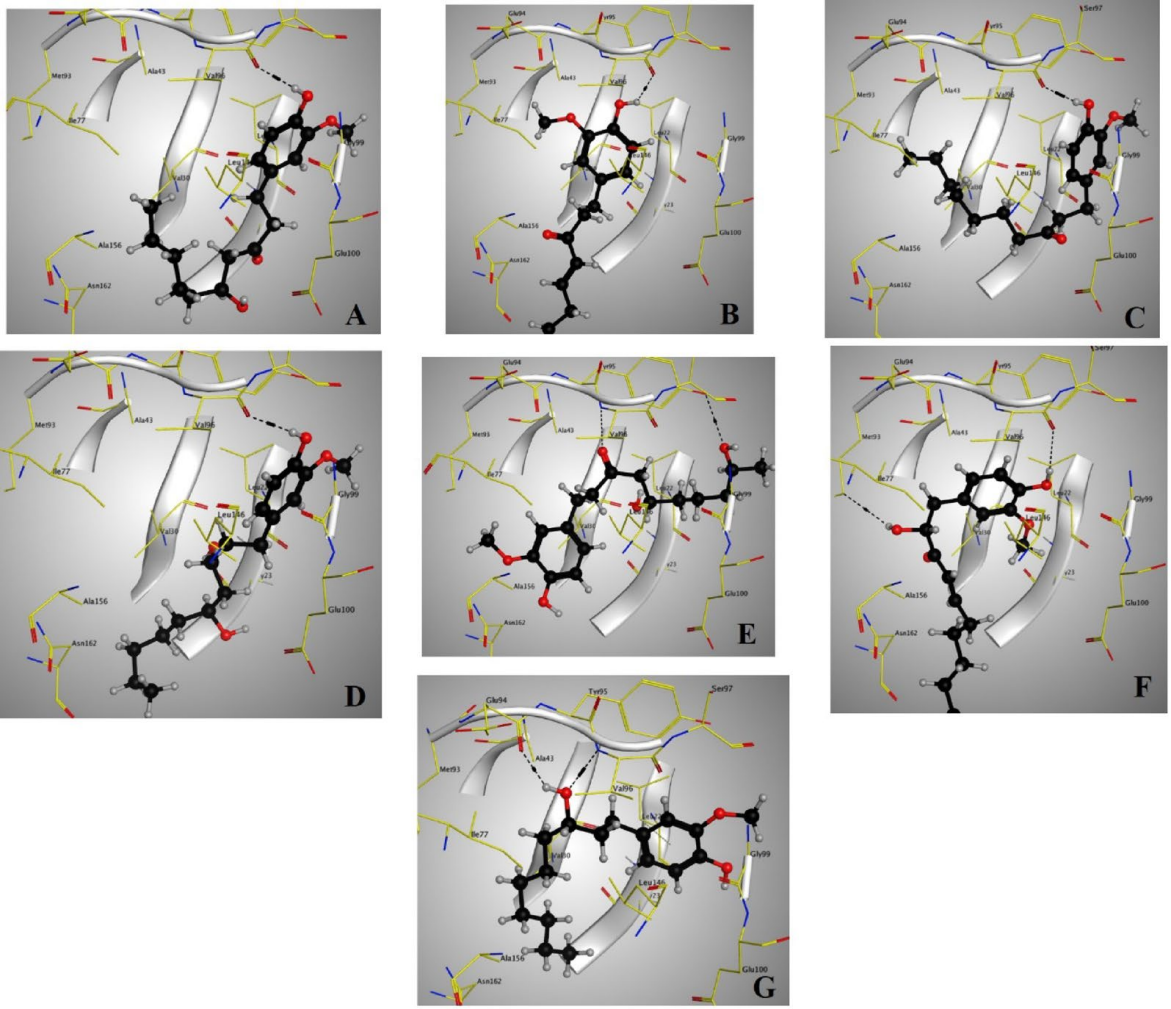
3D diagram of 6-gingerol (**A**), 6-shogaol (**B**), 6-paradol (**C**), 6-gingerdiol (**D**), hydroxylated-6-gingerol (**E**), hydroxylated-6-shogaol (**F**) and reduced paradol (**G**) interactions with AMPK binding site

6-Gingerdiol (M1) has the lowest binding score value (− 10.63 kcal/mol) among the tested compounds and is consequently expected to possess the highest nephroprotective effect. The docking between AMPK and 6-gingerdiol (Fig. 2D) was characterized by a constant present of a strong hydrogen bond (bond length of 2.92 Å) between aromatic hydroxyl group and Val96. Unlike 6-gingerol (Fig. 2A), both 3- and 5-hydroxyl groups of 6-gingerdiol were not involved in any interaction with the binding site.

Hydroxylated-6-gingerol (M2) showed the highest number of interactions among the tested compounds. Hydrogen bond was observed between 9-hydroxyl group and Tyr95, whereas the 3-carbonyl group formed two hydrogen bonds; one with Val96 and the other with Tyr95 (Fig. 2E).

(M2) has almost similar docking score as 6-gingerol, which indicated equivalent activity.

6-Shogaol was docked at the same site as 6-gingerdiol (Fig. 2B) but with a higher docking score, which revealed lower nephroprotective effect. Hydroxylated-6-shogaol (M3) exhibited H-bond interactions with Lys45, Met93 and Val96 (Fig. 2F). M3 showed a higher number of interactions with AMPK than the parent 6-shogaol. However, it displayed a higher docking score (− 8.73 kcal/mol), which revealed lower nephroprotective activity.

6-Paradol (M4), which resulted from the biotransformation of 6-shogaol with *B. subtilis* and *C. albicans*, showed higher nephroprotective activity than the parent compound 6- shogaol as evident by lower docking score value (− 8.99 kcal/mol) and stronger hydrogen bond (bond length of 2.84 Å) with the binding site (Fig. 2C).

Comparing reduced paradol (M5) with its parent 6-paradol, M5 showed lower docking score value (− 9.57 kcal/mol) and higher number of interactions with AMPK. It displayed H-bond interactions Glu94, Tyr95 and Vak96, as well as electrostatic interaction with Val96 (Fig. 2G). These results revealed the higher nephroprotective activity of M5 as compared to the parent 6-paradol.

## Discussion

The present study investigated the biotransformation potential of three microbial strains; *B. subtilis* 168, *P. aeruginosa* PO1A and *C. albicans* ATCC10231 on the phenolic compounds derived from *A. melegueta*; a plant rich in hydroxyphenylalkanes known for their diverse biological activities. Preliminary screening using TLC analysis demonstrated that *B. subtilis* could transform all three tested compounds, while *C. albicans* showed selective activity by modifying the structure of 6-shogaol. In contrast, *P. aeruginosa* did not produce detectable metabolites under the current experimental conditions. Traditional isolation and structural elucidation methods such as silica gel chromatography and spectroscopy generally require large sample volumes, making them less suitable for high-throughput screening. To overcome this limitation, LC–MS was employed, enabling rapid and sensitive detection and structural elucidation of metabolites resulting from the biotransformation based on accurate mass data. While MS/MS data and comparison with reported fragmentation profiles provided preliminary structural insights into the biotransformation products, we acknowledge that ^1^H and ^13^C NMR data are required for definitive structure confirmation. Due to the limited yield of certain metabolites, NMR analysis could not be performed at this stage. Scaling up for future isolation and full spectroscopic characterization is underway.

For the first time, the structural modification of *A. melegueta* phenolics was achieved via the enzymatic activity of *B. subtilis* and *C. albicans*. Biotransformation of 6-gingerol by *B. subtilis* yielded two major products: 6-gingerdiol and a hydroxylated derivative of 6-gingerol. Notably, 6-gingerdiol is reported here for the first time as a microbial metabolite, while the hydroxylated form has previously been observed via *Aspergillus niger*-mediated transformation (Takahashi et al. 1993). Similarly, *B. subtilis* transformed 6-shogaol into 6-paradol and hydroxylated 6-shogaol, both being reported here for the first time with this microorganism. Comparable transformations by *A. niger* (Jo et al. 2016; Lee 1995) suggest the presence of analogous enzymatic systems between the two species. Furthermore, 6-paradol was reduced to its corresponding reduced derivative by *B. subtilis*, a metabolite previously observed during in vitro rat liver biotransformation (Lee and Lee 1995). Collectively, these findings highlight the roles of reductase and hydroxylase enzymes in *B. subtilis* and its ability to structurally diversify hydroxyphenylalkanes.

Previous studies have demonstrated the nephroprotective effects of these phenolic compounds. For instance, 6-paradol has been shown to alleviate diclofenac (DIC)-induced acute kidney injury (AKI) through the activation of the AMPK/SIRT1 signaling pathway (Abdou et al. 2021). Likewise, Rodrigues et al. (2014) reported the nephroprotective effect of gingerol-rich fractions against gentamicin-induced nephropathy, and Han et al. (2019) found that 6-shogaol offers protection against ischemia-induced AKI. AMPK, a cellular energy regulator, is implicated in the pathophysiology of various renal disorders (Rajani et al. 2017). Interestingly, our prior findings indicated that DIC-induced AKI is associated with downregulation of AMPK/SIRT1 signaling pathway (Abdou et al. 2021), underscoring the therapeutic relevance of AMPK activation in nephropathy.

In this study, molecular docking was employed as an initial in silico approach to predict the possible nephroprotective potential of the biotransformed metabolites. This method is widely recognized in drug discovery as a valuable early screening tool for prioritizing active compounds and guiding further experimental validation (Sahu et al. 2024). However, experimental isolation and in vitro and in vivo nephroprotective testing of the metabolites remain essential for definitive assessment and recommended for future work. In this context, molecular docking was employed to assess the binding interactions of the parent compounds and their metabolites with AMPK. The docking results revealed that all tested compounds displayed favorable binding energies, suggesting potential nephroprotective properties. Among them, 6-gingerdiol exhibited the strongest binding affinity (lowest docking energy), indicating enhanced nephroprotective potential compared to its precursor, 6-gingerol. Similarly, 6-paradol showed greater binding affinity than 6-shogaol, and its reduced derivative demonstrated even higher predicted activity.

Microbial biotransformation not only diversifies the chemical space of naturally occurring phenolic compounds but may also enhance their biological efficacy. Modern biocatalysis employs microbial or enzyme-mediated reactions to generate structurally modified derivatives that are often difficult to access via conventional synthetic chemistry (e.g., reductive or hydroxylative pathways), offering a greener and more selective approach to novel bioactive molecule production. The fact that 6-gingerdiol has been identified for the first time as a microbial metabolite underscores the novelty of this approach and its potential to yield compounds with improved pharmacological profiles.

Furthermore, our in silico molecular docking against the AMPK protein predicted that all biotransformed metabolites bind with favorable energetics, with 6 gingerdiol exhibiting the strongest affinity, suggesting enhanced nephroprotective potential compared to its precursor. Numerous studies support similar approaches where molecular docking has successfully prioritized natural product derivatives for further evaluation. For example, flavonoids like baicalein and dihydromyricetin demonstrated strong docking scores against AMPK, consistent with their biological activation of the energy-sensing kinase (Moon 2024). These findings reinforce the concept that docking-based predictions can effectively guide experimental prioritization of promising lead compounds.

## Conclusion

To the best of our knowledge, this is the first report demonstrating the structural modification of 6-gingerol, 6-shogaol and 6-paradol through the biocatalytic activity of *B. subtilis* and *C. albicans*. Notably, 6-Gingerdiol was identified for the first time as a microbial transformation product. *B. subtilis* was also shown to modify the structure of *A. melegueta* major hyhroxyphenylalkanes via reductase and hydroxylase enzymes. Furthermore, molecular docking studies revealed higher nephroprotective activity for metabolites afforded by microbial biotransformation. These findings support the utility of microbial biotransformation in generating novel, bioactive derivatives with potentially superior therapeutic effects. They also emphasize the promise of using AMPK as a pharmacological target for evaluating nephroprotective agents through in silico approaches.

## Supplementary Information

Below is the link to the electronic supplementary material.

Supplementary Material 1

## Data Availability

All data supporting the findings of this study are included in the manuscript and/or its supplementary information files. Additional raw data is available from the corresponding author upon reasonable request.

## Abbreviations

AKI: Acute kidney injury
AMPK: AMP-activated protein kinase
DIC: Diclofenac
EDJ: 2-({5-Bromo-2-[(3, 4, 5-trimethoxyphenyl) amino]pyrimidin-4-yl}oxy)-N-methylbenzene-1-carboximidic acid
LC-TQ/MS: Liquid chromatography triple quadrupole mass spectrometry
MOE: Molecular operating environment
RMSD: Root mean square deviation
TLC: Thin layer chromatography

## References

[CR1] Abdou RM, El-Maadawy WH, Hassan M, El-Dine RS, Aboushousha T, El-Tanbouly ND, El-Sayed AM (2021) Nephroprotective activitiy of *Aframomum melegueta* seeds extract against diclofenac-induced acute kidney injury: a mechanistic study. J Ethnopharmacol. 10.1016/j.jep.2021.113939 33610709 10.1016/j.jep.2021.113939

[CR2] Alfarra HY, Omar MN (2012) Microbial transformation of natural products. Greener J Biol Sci 3(10):357–364

[CR3] Cichewicz RH, Kouzi SA (1998) Biotransformation of resveratrol to piceid by *Bacillus cereus*. J Nat Prod 61(10):1313–1314. 10.1021/np980139b 9784180 10.1021/np980139b

[CR4] Dugasani S, Pichika MR, Nadarajah VD, Balijepalli MK, Tandra S, Korlakunta JN (2010) Comparative antioxidant and anti-inflammatory effects of [6]-gingerol, [8]-gingerol, [10]-gingerol and [6]-shogaol. J Ethnopharmacol 3(2):515–520. 10.1016/j.jep.2009.10.004 10.1016/j.jep.2009.10.004 19833188

[CR5] El-Halawany AM, Dine EL, RS, El Sayed NS, Hattori M (2014) Protective effect of *Aframomum melegueta* phenolics against CCl 4 -induced rat hepatocytes damage; Role of apoptosis and pro-inflammatory cytokines inhibition. Sci Rep 4:1–9. 10.1038/srep05880 10.1038/srep05880 PMC5376205 25077538

[CR6] Gaire BP, Kwon OW, Park SH, Chun KH, Kim SY, Shin DY, Choi JW (2015) Neuroprotective effect of 6-paradol in focal cerebral ischemia involves the attenuation of neuroinflammatory responses in activated microglia. PLoS ONE 10(3):e0120203. 10.1371/journal.pone.0120203 25789481 10.1371/journal.pone.0120203 PMC4366308

[CR7] Gentile DM, Tomlinson ES, Maggs JL, Park BK, Back DJ (1996) Dexamethasone metabolism by human liver in vitro. Metabolite identification and inhibition of 6-hydroxylation. J Pharmacol Exp Ther 277(1):105–112 8613906

[CR8] Guo J, Wu H, Du L, Zhang W, Yang J (2014) Comparative antioxidant properties of some gingerols and shogaols, and the relationship of their contents with the antioxidant potencies of fresh and dried ginger (*Zingiber officinale* Roscoe). J Agric Sci Technol 16(5):1063–1072

[CR9] Han SJ, Kim M, D’Agati VD, Lee HT (2019) 6-shogaol protects against ischemic acute kidney injury by modulating NF-κB and heme oxygenase-1 pathways. Am J Physiol-Renal Physiol 317(3):F743–F756. 10.1152/ajprenal.00182.2019 31313953 10.1152/ajprenal.00182.2019 PMC6766624

[CR10] Jo SK, Kim IS, Rehman SU, Ha SK, Park HY, Park YK, Yoo HH (2016) Characterization of metabolites produced from the biotransformation of 6-shogaol formed by *Aspergillus niger*. Eur Food Res Technol 242(1):137–142

[CR11] Koh IK, Lee SS (1983) Biodegradation mechanism of shogaol by *Aspergillus niger*. Yakhak Hoeji 27(1):29–36

[CR12] Kou X, Wang X, Ji R, Liu L, Qiao Y, Lou Z, Ma C, Li S, Wang H, Ho CT (2018) Occurrence, biological activity and metabolism of 6-shogaol. Food Funct 9(3):1310–1327. 10.1039/c7fo01354j 29417118 10.1039/c7fo01354j

[CR13] Kumara M, Shylajab MR, Nazeemc P, Babu T (2017) 6-Gingerol is the most potent anticancerous compound in ginger *Zingiber officinale Rosc*. J Dev Drugs 6(1):167. 10.4172/2329-6631.1000167

[CR14] Lee S (1995) Re-examination of 6-shogaol biotransformation by *Aspergillus niger*. Arch Pharm Res 18(2):136–137

[CR15] Lee SS, Lee WY (1995) Biotransformation of dehydroparadols by *Aspergillus niger*. Arch Pharm Res 18(6):458–461. 10.1007/BF02976352

[CR16] Moon DO (2024) Plant-derived flavonoids as AMPK activators: unveiling their potential in type 2 diabetes management through mechanistic insights, docking studies, and pharmacokinetics. Appl Sci 14(19):8607. 10.3390/APP14198607

[CR17] Pan MH, Hsieh MC, Kuo JM, Lai CS, Wu H, Sang S, Ho CT (2008) 6-shogaol induces apoptosis in human colorectal carcinoma cells via ROS production, caspase activation, and GADD 153 expression. Mol Nutr Food Res 52(5):527–537. 10.1002/mnfr.200700157 18384088 10.1002/mnfr.200700157

[CR18] Rajani R, Pastor-Soler NM, Hallows KR (2017) Role of AMP-activated protein kinase in kidney tubular transport, metabolism, and disease. Curr Opin Nephrol Hypertens 26(5):375–383. 10.1097/MNH.0000000000000349 28614117 10.1097/MNH.0000000000000349

[CR19] Rodrigues FAP, Prata MMG, Oliveira ICM, Alves NTQ, Freitas REM, Monteiro HSA, Silva JA, Vieira PC, Viana DA, Libório AB, Havt A (2014) Gingerol fraction from *Zingiber officinale* protects against gentamicin-induced nephrotoxicity. Antimicrob Agents Chemother 58(4):1872–1878 24395230 10.1128/AAC.02431-13 PMC4023749

[CR20] Sahu D, Rathor LS, Dwivedi SD, Shah K, Chauhan NS, Singh MR, Singh D (2024) A review on molecular docking as an interpretative tool for molecular targets in disease management. Assay Drug Dev Technol 22(1):40–50. 10.1089/ADT.2023.060 38232353 10.1089/adt.2023.060

[CR21] Sapkota A, Park SJ, Choi JW (2019) Neuroprotective effects of 6-shogaol and its metabolite, 6-paradol, in a mouse model of multiple sclerosis. Biomol Ther (Seoul) 1(2):152–159 10.4062/biomolther.2018.089 PMC6430232 30001610

[CR22] Seow SLS, Hong SL, Lee GS, Malek SNA, Sabaratnam V (2017) 6-shogaol, a neuroactive compound of ginger (jahe gajah) induced neuritogenic activity via NGF responsive pathways in PC-12 cells. BMC Complement Altern Med 17(1):334. 10.1186/s12906-017-1837-6 28646880 10.1186/s12906-017-1837-6 PMC5483314

[CR23] Shah SAA, Tan HL, Sultan S, Faridz MABM, Shah MABM, Nurfazilah S, Hussain M (2014) Microbial-catalyzed biotransformation of multifunctional triterpenoids derived from phytonutrients. Int J Mol Sci 15(7):12027–12060. 10.3390/ijms150712027 25003642 10.3390/ijms150712027 PMC4139828

[CR24] Takahashi H, Hashimoto T, Noma Y, Asakawa Y (1993) Biotransformation of 6-gingerol and 6-shogaol. Phytochemisry 34(6):1497–1500

[CR25] Tian L, Qian W, Qian Q, Zhang W, Cai X (2020) Gingerol inhibits cisplatin-induced acute and delayed emesis in rats and minks by regulating the central and peripheral 5-HT, SP, and DA systems. J Nat Med 74(2):353–370. 10.1007/s11418-019-01372-x 31768887 10.1007/s11418-019-01372-x PMC7044144

[CR26] Venisetty R, Ciddi V (2005) Application of microbial biotransformation for the new drug discovery using natural drugs as substrates. Curr Pharm Biotechnol 4(3):153–167 10.2174/1389201033489847 12769760

[CR27] Wang S, Zhang C, Yang G, Yang Y (2014) Biological properties of 6-gingerol: a brief review. Nat Prod Commun 9(7):1027–1030. 10.1177/1934578x1400900736 25230520

[CR28] Young HY, Luo YL, Cheng HY, Hsieh WC, Liao JC, Peng WH (2005) Analgesic and anti-inflammatory activities of [6]-gingerol. J Ethnopharmacol 4:207–210 10.1016/j.jep.2004.09.009 15588672

