# Supplementary material for: In silico nephroprotective evaluation of microbial biotransformed metabolites from Aframomum melegueta
Source: AMB Express. 2025 Oct 22;15:154. doi: 10.1186/s13568-025-01962-x (PMC12546235; doi:10.1186/s13568-025-01962-x)
Supplement: Supplementary file 1 — Supplementary Material 1 [file 13568_2025_1962_MOESM1_ESM.docx]

**Microbial biotransformation of *Aframomum melegueta* phenolics and nephroprotective activity evaluation**

Rabab M. Abdou^1*^, Riham S. El-Dine^1^, Reham Samir^2^, Nebal D. El-Tanbouly^1^, Aly M. El-Sayed^1^

^1^ Department of Pharmacognosy, Faculty of Pharmacy, Cairo University, Kasr El-Aini Street, Cairo, 11562, Egypt

^2^ Department of Microbiology and Immunology Pharmacognosy, Faculty of Pharmacy, Cairo University, Kasr El-Aini Street, Cairo, 11562, Egypt

Corresponding author email: rabab.abdo@pharma.cu.edu.eg

**LC-TQMS analysis for** **biotransformation medium samples of *A. melegueta* K. schum seeds phenolics**

LC-MS analysis was carried out in the Center for Drug Discovery Research and Development, Faculty of Pharmacy, Ein-Shams University, Egypt, using XEVO TQD triple quadrupole instrument equipped with ACQUITY UPLC-BEH C^18^ (1.7 µm- 2.1×50 mm) column and Waters Corporation mass spectrometer (Milford, MA01757, USA). The sample was dissolved in HPLC-grade methanol (100 μg/mL), filtered using a membrane disc filter (0.2 μm) then injected (10 μL) into the UPLC instrument. Mobile phase was filtered through 0.2 μm filter membrane disc and degassed by sonication before injection. It consisted of H_2_O acidified with 0.1% formic acid (A) and methanol acidified with 0.1% formic acid (B) at 0.2 mL/min flow rate. Elution was performed using the following gradient program: 0-2 min, 10% B; 2-5 min, 10-30% B; 5-15 min, 30-70% B; 15-22 min, 70-90% B; 22-25 min, 90% B; 25-26 min, 90-100% B; 26-29 min, 100% B and then 10% B for 3 mins to equilibrate the column.

The parameters for analysis were carried out using negative ion mode as follows: source temperature 150 °C, cone voltage 30 eV, capillary voltage 3 kV, desolvation temperature 440 °C, cone gas flow 50 L/h, and desolvation gas flow 900 L/h. Mass spectra were detected in the ESI between *m/z* 100–1000. The peaks and spectra were processed using the Maslynx 4.1 software.

**Table S****1 Type of interactions of *A. melegueta* K. schum** **phenolics and their metabolites with amino acids of AMP-activated protein kinase**

| **Compound** | **Amino acids** | **Interacting groups** | **Type of interaction** | **Bond length** |
| --- | --- | --- | --- | --- |
| **EDJ** | Met93  Glu94  Val96  Val96  Val96 | Br  CH (Phenyl)  N (Pyrimidine)  NH  CH (Phenyl) | Halogen bond  Electrostatic  H-bond acceptor  H-bond donor  Electrostatic | 3.84  3.24  3.12  2.97  3.26 |
| **6-Gingerol*** | Val96  Glu100 | OH  OH | H-bond donor  H-bond donor | 3.00  3.33 |
| **6-Gingerdiol (M1)** | Val96 | OH | H-bond donor | 2.92 |
| **Hydroxylated-6-Gingerol (M2)** | Met93  Tyr95  Tyr95  Val96 | CH_3_  OH  O (C=O)  O (C=O) | Electrostatic  H-bond acceptor  H-bond acceptor  H-bond acceptor | 3.85  3.03  3.21  3.24 |
| **Shogaol*** | Val96 | OH | H-bond donor | 3.06 |
| **Hydroxylated-6-Shogaol (M4)** | Lys45  Met93  Val96 | O (C=O)  OH  OH | H-bond acceptor  H-bond donor  H-bond donor | 3.15  3.63  3.55 |
| **6-Paradol *(M3)** | Val96 | OH | H-bond donor | 2.84 |
| **Reduced Paradol (M5)** | Glu94  Tyr95  Vak96  Val96 | OH  OH  OH  CH (Phenyl) | H-bond donor  H-bond acceptor  H-bond acceptor  Electrostatic | 2.93  3.41  3.00  3.50 |

***** Parent compound

**
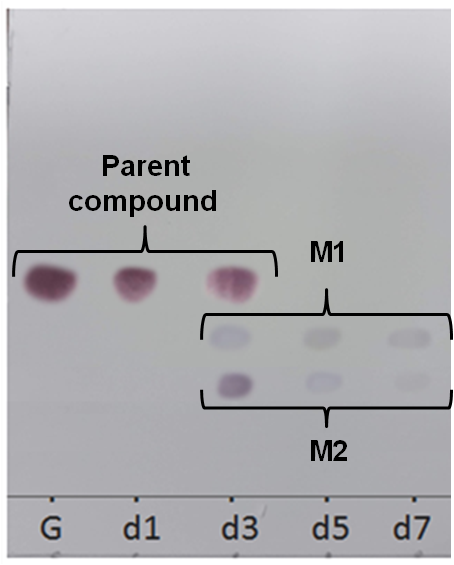
**

**Fig. S1** TLC monitoring of **6-gingerol** biotransformation by ***Bacillus subtilis***


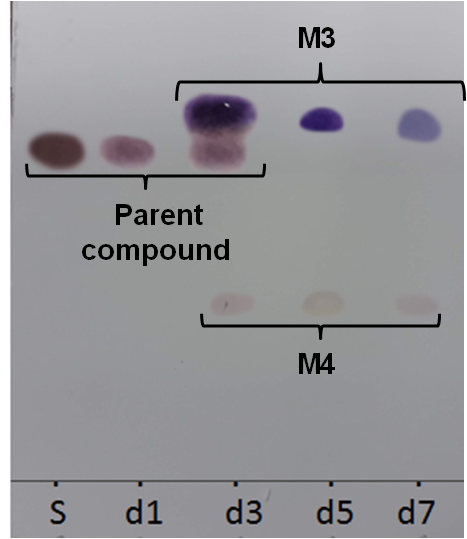


**Fig. S2** TLC monitoring of **6-shogaol** biotransformation by ***Bacillus subtilis***


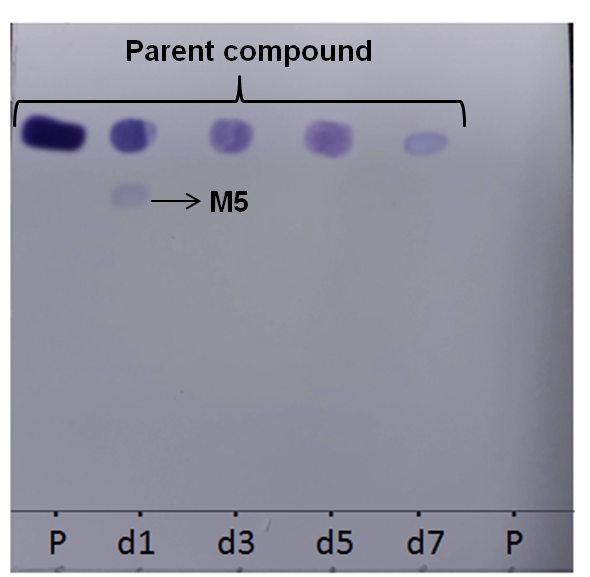


**Fig. S3** TLC monitoring of **6-paradol** biotransformation by ***Bacillus subtilis***

**
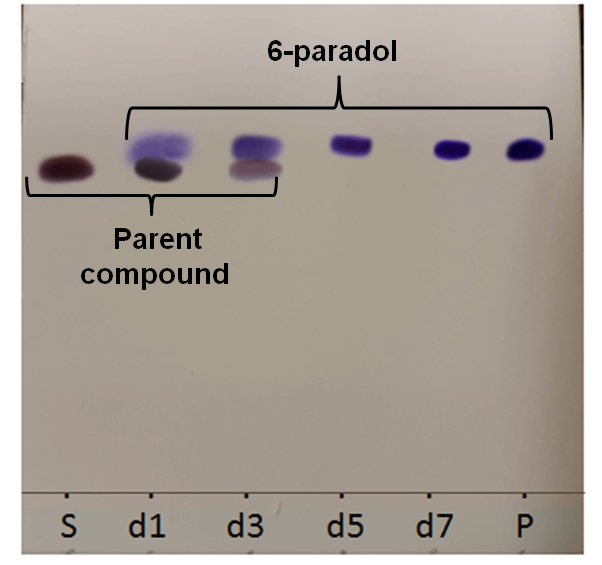
**

**Fig. S4** TLC monitoring of **6-shogaol** biotransformation by ***Candida albicans***


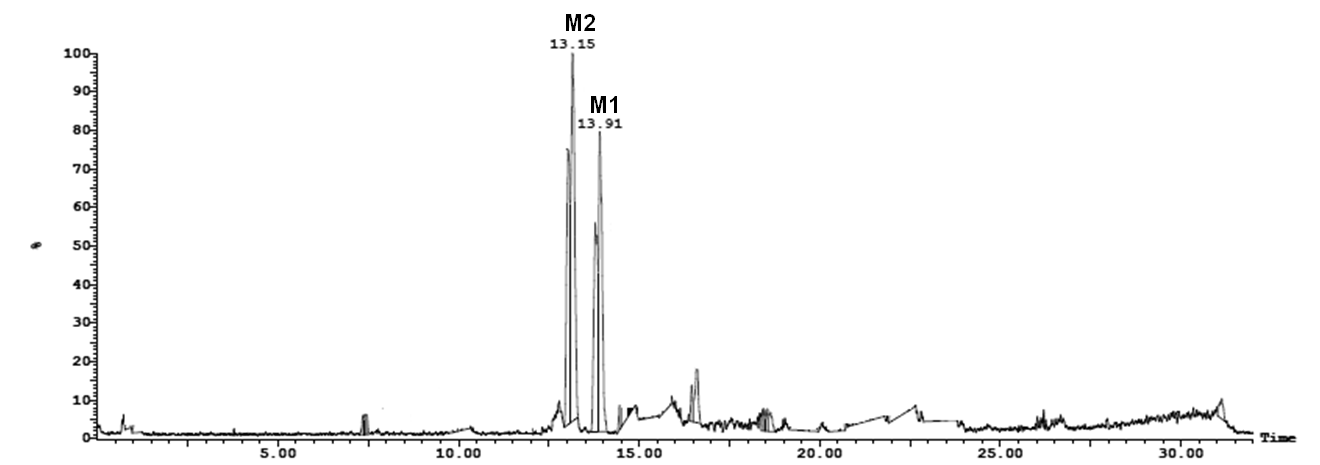

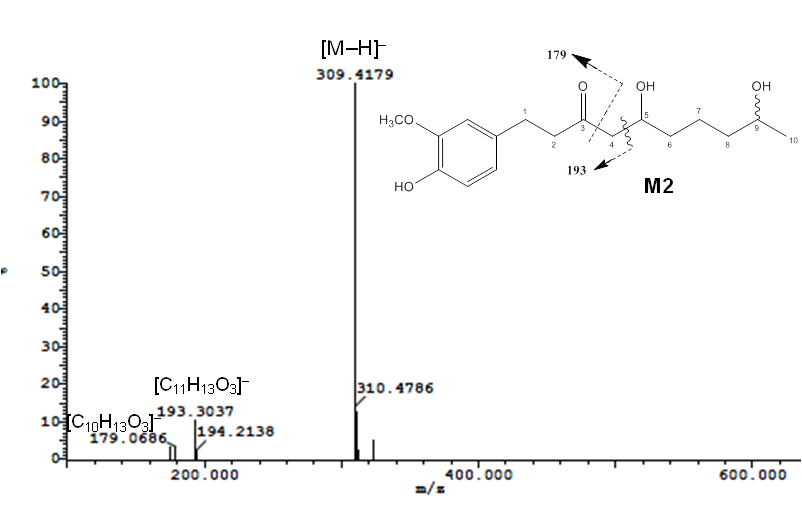

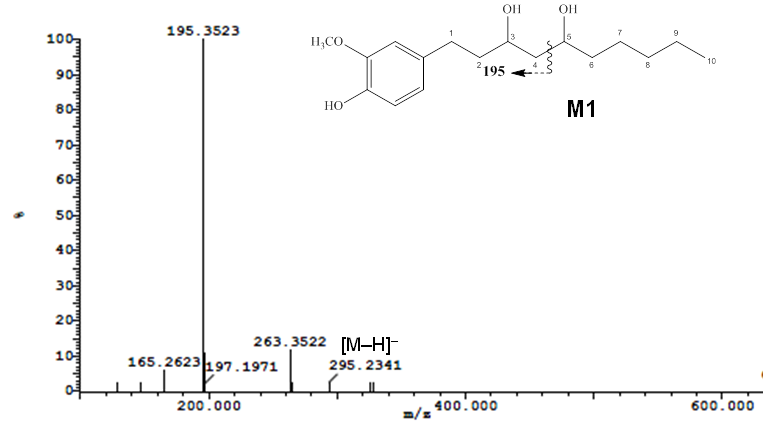


**A**

**C**

**B**

**Fig. S5** Representative LC-MS total ion chromatogram for metabolites in the reaction sample of 6-gingerol with *B. subtilis* 168 (A) and MS/MS spectra of M1- M2 (B and C, respectively).


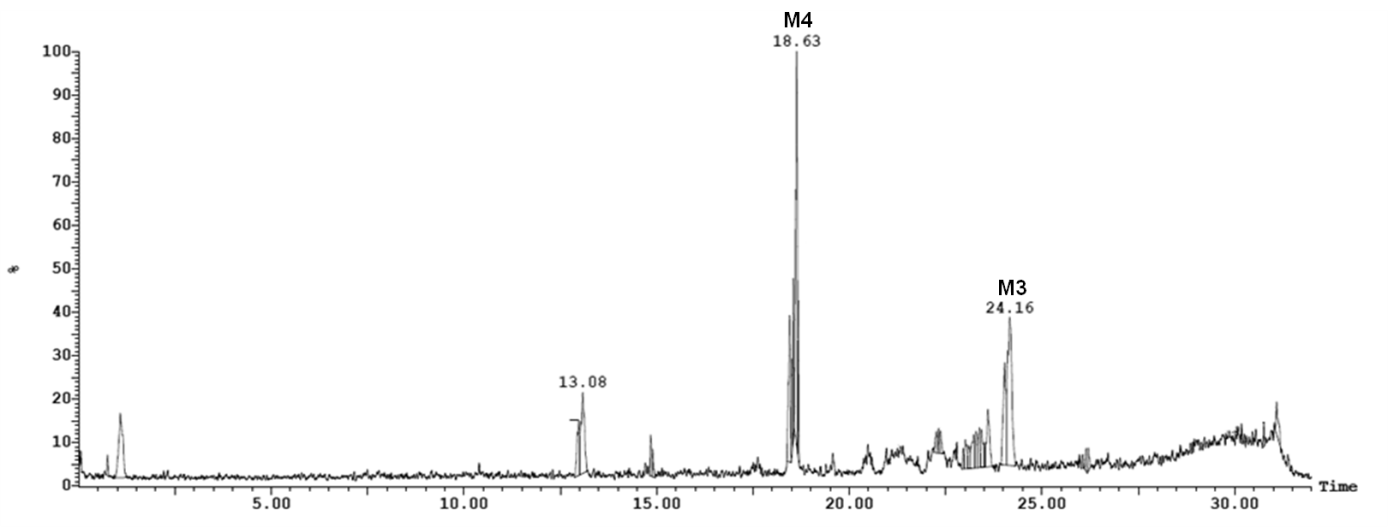

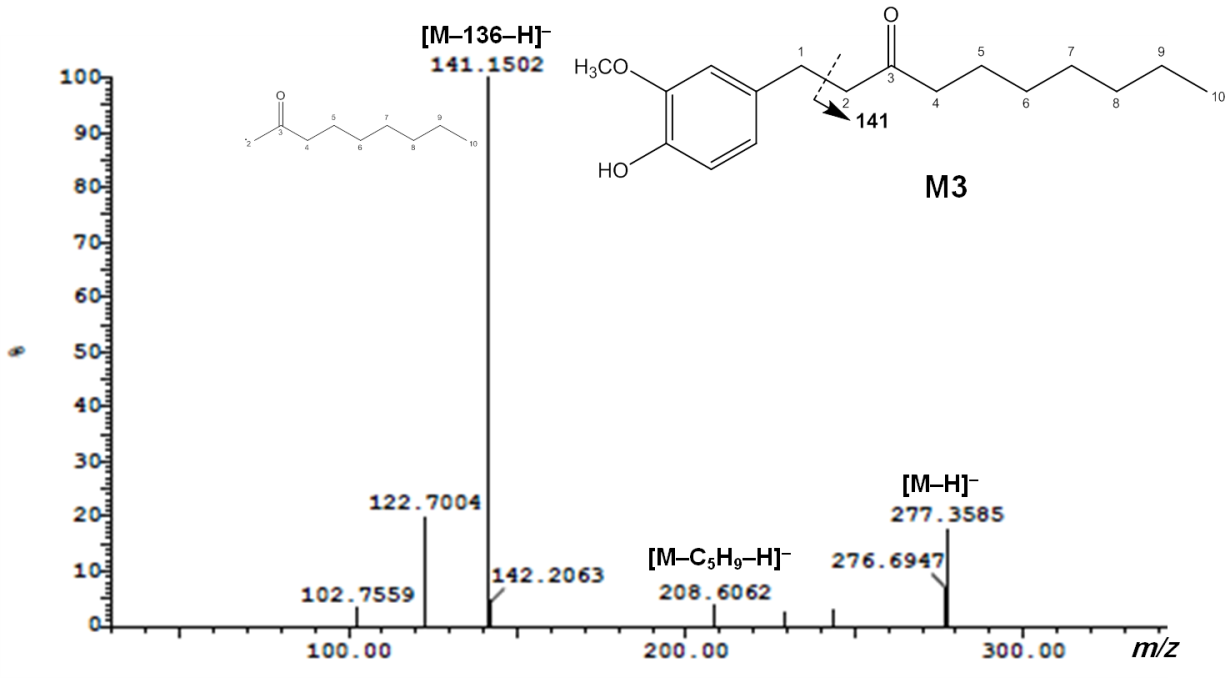

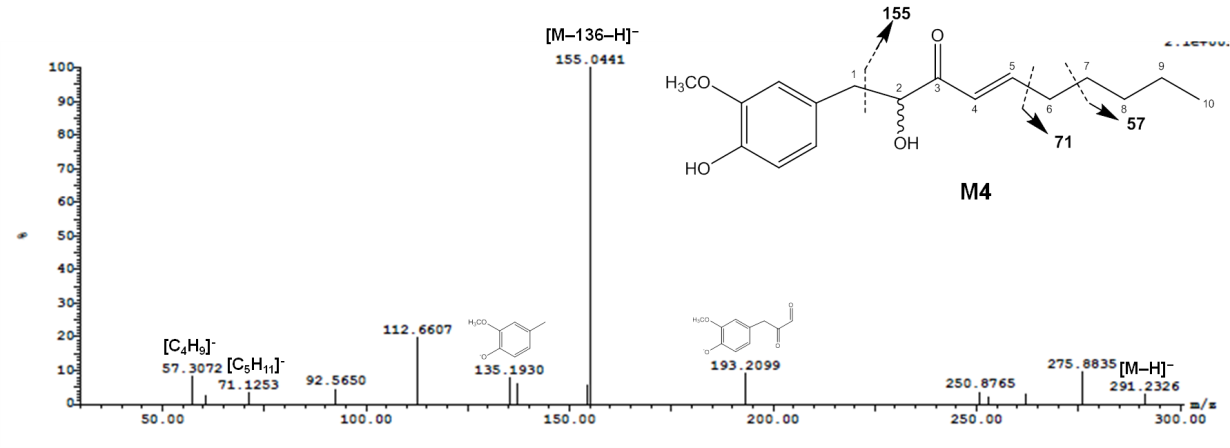


**A**

**B**

**C**

**Fig. S6** Representative LC-MS total ion chromatogram for metabolites in the reaction sample of 6-shogaol with *B. subtilis* 168 (A) and MS/MS spectra of M3-M4 (B and C, respectively).


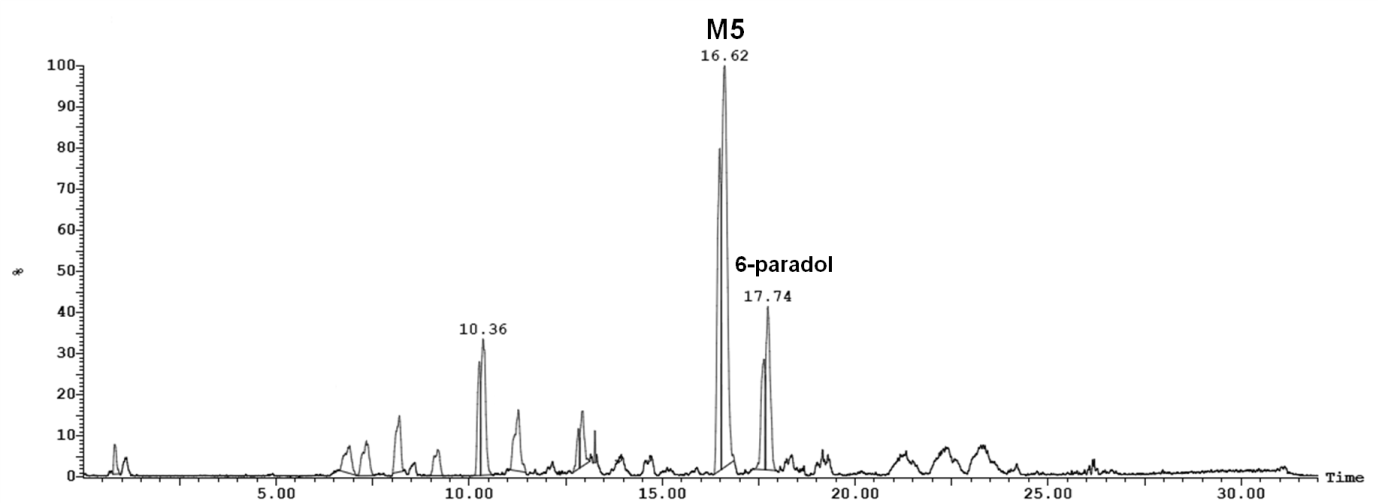

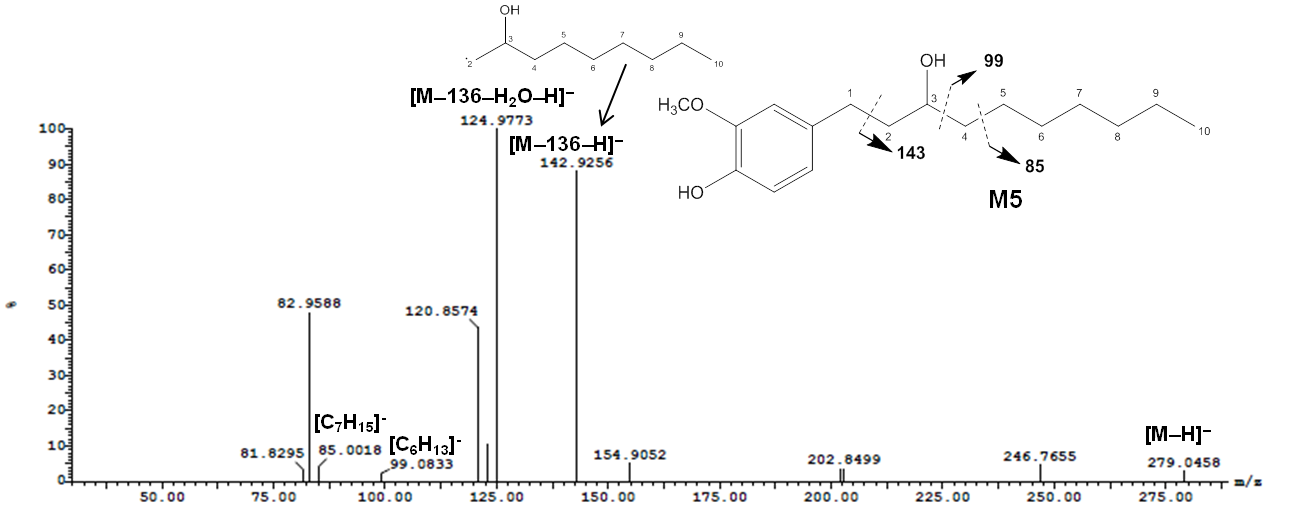


**A**

**B**

**Fig. S7** Representative LC-MS total ion chromatogram for metabolites in the reaction sample of 6-paradol with *B. subtilis* 168 (A) and MS/MS spectra of M5 (B).


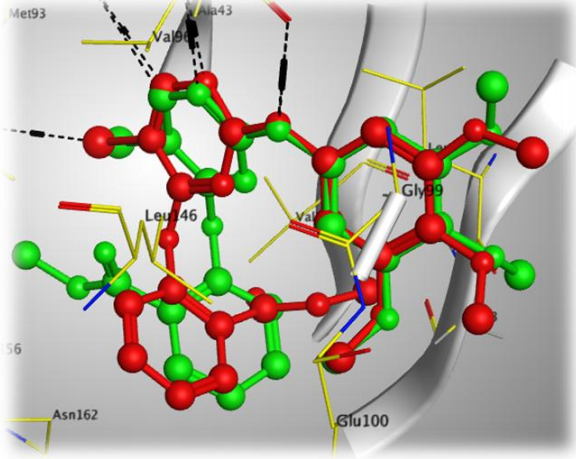


**Fig. S8** 3D representation of the superimposition of the co-crystallized (red) and

the docking pose (green) of EDJ in the active site of AMPK
